# A Nanofibrillated Cellulose-Based Electrothermal Aerogel Constructed with Carbon Nanotubes and Graphene

**DOI:** 10.3390/molecules25173836

**Published:** 2020-08-24

**Authors:** Bing Zhuo, Shuoang Cao, Xinpu Li, Jiahao Liang, Zhihong Bei, Yutong Yang, Quanping Yuan

**Affiliations:** School of Resources, Environment and Materials, Guangxi University, Nanning 530004, China; zhuob320@163.com (B.Z.); shuoang1997@163.com (S.C.); lixinpu0517@163.com (X.L.); jiahooliang@163.com (J.L.); beizh0310@163.com (Z.B.); yyt5919@163.com (Y.Y.)

**Keywords:** cellulose, aerogel, electrothermal composite, carbon nanotubes, graphene

## Abstract

Nanofibrillated cellulose (NFC) as an environmentally friendly substrate material has superiority for flexible electrothermal composite, while there is currently no research on porous NFC based electrothermal aerogel. Therefore, this work used NFC as a skeleton, combined with multi-walled carbon nanotubes (MWCNTs) and graphene (GP), to prepare NFC/MWCNTs/GP aerogel (CCGA) via a simple and economic freeze-drying method. The electrothermal CCGA was finally assembled after connecting CCGA with electrodes. The results show that when the concentration of the NFC/MWCNTs/GP suspension was 5 mg mL^−1^ and NFC amount was 80 wt.%, the maximum steady-state temperature rise of electrothermal CCGA at 3000 W m^−2^ and 2000 W m^−2^ was of about 62.0 °C and 40.4 °C, respectively. The resistance change rate of the CCGA was nearly 15% at the concentration of 7 mg mL^−1^ under the power density of 2000 W m^−2^. The formed three-dimensional porous structure is conducive to the heat exchange. Consequently, the electrothermal CCGA can be used as a potential lightweight substrate for efficient electrothermal devices.

## 1. Introduction

Low-dimensional nano-carbon materials, such as one-dimensional tubular carbon nanotubes (CNTs) and two-dimensional lamellar graphene (GP) are currently widely used in the preparation of nano-functional composites for their unique physical and mechanical properties [1,2]. Both CNTs and GP have Young’s modulus of ~1 TPa [3,4]. Due to their excellent electrical conductivity and thermal conductivity [5,6,7,8], they were involved in many researches on functional materials. Of which, electrothermal functional composite can convert electrical energy into thermal energy on the basis of Joule heating principle principally due to the inelastic collision of charged particles under the electric field [9]. Recently, related studies reported various electrothermal composites made of CNTs or GP, which can be used for medical infusion apparatus [10], deicing [11,12], defrosting and defogging [13,14,15], repairing and self-curing resin [16], and other fields [17,18,19,20]. As example, a fluoroalkyl-silane-modified three-dimensional graphene foam composite (FS-GF) with a porous structure was fabricated, which can be used in the Joule heating mesh for airflow; the prepared FS-GF also demonstrated rapid heating rate, high conversion efficiency, and uniform temperature distribution [21]. Moreover, a porous aramid nanofiber/CNT electrothermal hybrid aerogel film coated with a layer of fluorocarbon resin exhibited fast heating speed and reliable cycle stability [22]. The fast heating speed was attributed to that there were two heat dissipation pathways in the porous aerogel, such as heat conduction and heat convection [23]. CNTs and GP had been proved to have some advantages in the application of electric heating, and were expected to replace many traditional electrothermal composites [24]. They are also considered as the candidate materials for the preparation of ultralight, elastic, and conductive aerogels [25], presenting porous network structure and large specific surface area [23,26].

There are not many reports about the electrothermal aerogels, except for the existed reduced graphene oxide (rGO) electrothermal aerogels [23]. However, functional aerogels also represent limitations, such as that their mechanical properties remain to be further enhanced [27]. And the method of combination with polymer was also existed, such as the poly(methyl methacrylate) [21] and aramid fiber [22] has been reported, but the used polymer is not easy to degrade. Besides, the nano-carbon materials are randomly stacking due to their large van der Waals forces and poor dispersion stability in water [28]. This fact leads to the nano-carbon materials having difficultly-uniform dispersion in polymers [29]. Thus, many preparation methods employed chemical modification, by which some properties of nano-carbon materials would be weakened; such as that covalent modification can change the inherent properties of CNTs and graphene to a certain extent, resulting in the loss of the conductivity property [30]. Moreover, non-covalent modification can improve the dispersion effect under the action of surfactants, but the modification with a high amount of surfactant will impair the mechanical properties of the composites prepared by compounding polymers as matrix [31]. Consequently, how to avoid the agglomeration of nano-carbon materials in substrate is always particularly important for improving the performance of their composites [32].

Nanofibrillated cellulose (NFC) is a kind of nanocellulose, and generally used as a dispersant and binder [33,34]. It is also a biodegradable, renewable, easily available, and environmentally-friendly natural polymer [35,36]. Many studies have shown that NFC can be used as a dispersant to promote dispersion stability of hydrophobic CNTs and rGO in aqueous suspension [37,38]. Meanwhile, NFC can improve the mechanical properties of its composite [39]. For example, the mechanical strength of a CNFs/rGO film was significantly improved as compared with that of pure rGO film [40].

In the past, researches focused on the preparation of NFC-based electrothermal composite films with flexibility [41,42,43], while there was rare research on the assembly of porous NFC-based electrothermal aerogel. In this work, a porous electrothermal NFC/MWCNTs/GP aerogel (CCGA) was presented, which was prepared via a simple and effective ultrasonic dispersion and freeze-drying method. The work mainly focused on the influence of ultrasonic parameters, suspension concentration, and NFC content on its electric heating performance and other properties. This research will provide a reference for the subsequent improvement of the preparation process, as well as for enhancing surface heat exchange of the other electrothermal composites.

## 2. Materials and Methods

### 2.1. Materials

The initial NFC suspension (mass concentration of 1.08 wt.%, the diameter of 10–20 nm) was purchased from Tianjin Woodelf Biotechnology Co., Ltd., Tianjin, China, and made from bleached kraft pulp of spruce wood; the preparation process of the chemical TEMPO oxidation, wash, and high-pressure homogenization was employed. Single-layer graphene (purity > 99%, sheet diameter 0.5–5 µm, and a specific surface area 1000–1217 m^2^ g^−1^) and industrial-grade MWCNTs powder was produced by Suzhou Tanfeng Graphene Technology Co., Ltd., Suzhou, China.

### 2.2. Methods

#### 2.2.1. Preparation of CCGA with Different Ultrasonic Time and Power

In this section, CCGA with 60 wt.% NFC was prepared, and the mass ratio between MWCNTs and GP in the CCGA was 7:3. Firstly, NFC suspension of 2.780 g was weighed and added into a 50 mL beaker; along with distilled water was poured in the beaker to prepare 5 mg mL^−1^ NFC suspension via ultrasonic cell disruptor (TL–1200Y, Jiangsu Tenlin Instrument Co. Ltd., Yancheng, China). Ultrasonic power of 1000 W and time of 10 min was employed.

Next, 0.014 g MWCNTs was weighed and then added into the above NFC suspension to prepare NFC/MWCNTs suspension with the help of ultrasonic treatment (1000 W, 10 min).

GP suspension was prepared respectively. After distilled water was poured into a 50 mL beaker in which 0.006 g graphene was added in advance, GP suspension with a concentration of 1 mg mL^−1^ was prepared firstly using a magnetic stirrer with a speed of 1000 r min^−1^ for 5 min, then sonicated at 1000 W for 10 min.

**Figure 1:**
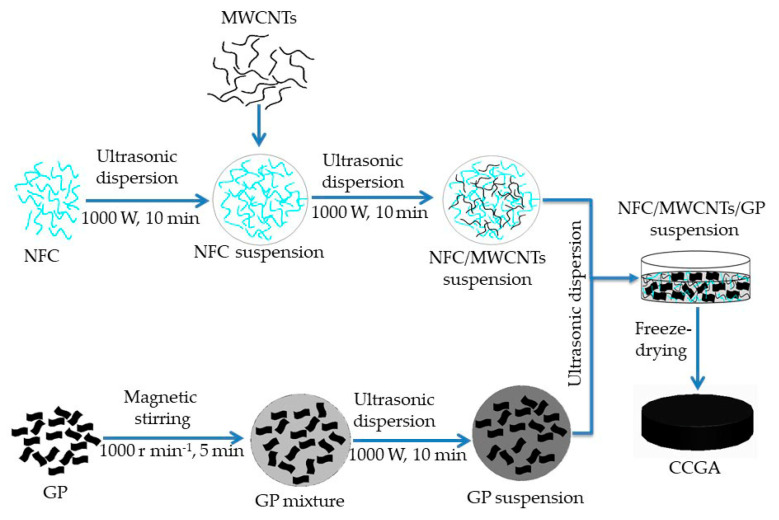
Scheme for preparation of the nanofibrillated cellulose/multi-walled carbon nanotubes/graphene aerogel (NFC/MWCNTs/GP) (CCGA).

Finally, the prepared GP suspension was poured into the NFC/MWCNTs suspension to prepare NFC/MWCNTs/GP suspension with a concentration of 5 mg mL^−1^ via following ultrasonic treatments. Various ultrasonic powers (600 W, 800 W, 1000 W) was carried out at the controlled ultrasonic time of 40 min, as well as that different ultrasonic time (10 min, 30 min, 50 min, 70 min) under the fixed ultrasonic power of 1000 W. The obtained suspension was then poured into a flat-bottom petri dish (diameter of 55 mm) before it was put into a vacuum freeze dryer (FD–1A–50, Jiangsu Tenlin Instrument Co., Ltd., Yancheng, China). After it froze at a temperature of about –50 °C for 2 h and was vacuum freeze-dried for 48 h, the CCGA was obtained. The preparation process of the CCGA is shown in Figure 1. The macroscopic morphology of CCGA was observed using a camera. Finally, the electric heating performance was tested.

#### 2.2.2. Preparation of CCGA with Different Suspension Concentrations

According to the above 2.2.1 preparation process, different concentrations (3 mg mL^−1^, 4 mg mL^−1^, 5 mg mL^−1^, 6 mg mL^−1^, 7 mg mL^−1^) of NFC/MWCNTs/GP suspension with total volume of 12 mL were prepared under the fixed ultrasonic power of 1000 W for 50 min. Then they were freeze-dried to be the CCGA. CCGA prepared from different suspension concentrations were respectively recorded as x–CCGA (where x represents different concentrations of 3 mg mL^−1^, 4 mg mL^−1^, 5 mg mL^−1^, 6 mg mL^−1^, 7 mg mL^−1^). Their electric heating performance was tested.

#### 2.2.3. Preparation of CCGA with Different NFC Contents

Similarly, according to the above 2.2.1 and 2.2.2 preparation process, CCGA with different amount of NFC (30 wt.%, 40 wt.%, 50 wt.%, 60 wt.%, 70 wt.%, 80 wt.%), and the controlled suspension concentration of 5 mg mL^−1^, which were denoted as CCGA y (where y represents the amount of NFC accounted for CCGA (30 wt.%, 40 wt.%, 50 wt.%, 60 wt.%, 70 wt.%, 80 wt.%). Their morphology, phase structure, chemical property, and thermal stability were characterized, as well as their electric heating performance. Moreover, pure NFC suspension and the mixture suspensions with various NFC contents (CCGA 40, CCGA 60, and CCGA 80) were prepared according to the Section 2.2.1 above. After the obtained suspensions were cooled to room temperature, the pH values were measured in the environment with constant temperature using pH meter (testo 206–ph–1, Testo Instruments (Shenzhen) Co., Ltd., Shenzhen, China), and electrical conductivity of the suspensions were also measured via the conductivity meter (DS–307A, INESA Scientific Instrument Co., Ltd., Shanghai, China).

### 2.3. Characterization

Morphology of CCGA with various NFC amounts was characterized by scanning electron microscope (SEM; S–3400N, Hitachi, Chiyoda–ku, Japan). X-ray diffraction (XRD; SmartLab 3 kW, Rigaku Corp., Akishima–shi, Japan) were carried out on CCGA, NFC, MWCNTs at the condition of 2*θ* from 5° to 70° with a scanning speed of 8° min^−1^. Fourier transform infrared spectra (FT-IR) of powder samples of CCGA, NFC, MWCNTs, and GP, were collected from 400 to 4000 cm^−1^ with a Nicolet iS50 spectrometer (Thermo Fisher Scientific, Waltham, MA, USA) that has a resolution better than 0.09 cm^−1^. The thermal stability of the samples was analyzed by a differential thermal-thermogravimetric synchronous analyzer (DTA–TG; DTG–60 (H), Shimadzu Corp., Kyoto, Japan) in a nitrogen atmosphere from room temperature to 600 °C with a temperature rise rate of 10 °C min^−1^. As well as analysis on the thermal stability of NFC/MWCNTs aerogel (NFC content was 60 wt.%) prepared using the above process was also executed for the purpose of comparison with the above samples.

### 2.4. Preparation of Electrothermal CCGA and Test for Electric Heating Performance

**Figure 2:**
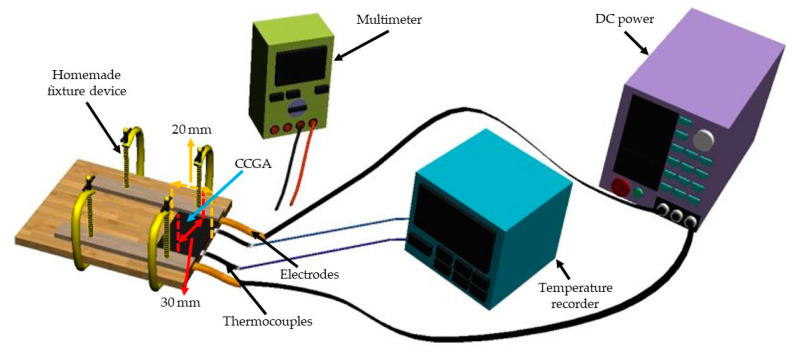
Test device for electric heating performance.

As shown in Figure 2, the prepared CCGA samples with various suspension concentrations were cut into 20 × 40 mm^2^ (effective area of 20 × 30 mm^2^), with copper foil (thickness 0.02 mm, width 5 mm) as electrodes attached to both ends of them. And they were fixed by a clamping device. At first, the resistance between two electrodes was tested using a Fluke 15B+ multimeter (Fluke Corp., Washington, DC, USA). After calculating the input voltage on the basis of Joule’s law and the set power density, the input voltage was supplied by a DC constant voltage power (MESTEK DP605C; Shenzhen MESTEK Tools Co. Ltd., Shenzhen, China) which can record the voltage and electric current during the electrification, and a multi-channel temperature recorder (SIN–R960, Hangzhou Sinomeasure Automation Technology Co. Ltd., Hangzhou, China) was used to record temperature during the test process. Temperature distribution on the CCGA during the electrification process was observed using a thermal infrared imager (TiS40, Fluke Corp., Washington, DC, USA).

#### 2.4.1. Electrothermal CCGA Prepared with the Different Ultrasonic Treatments

After the electrothermal CCGA with different ultrasonic treatments were assembled, the temperature distribution on the CCGA was obtained using the thermal infrared imager under the power density of 1000 W m^−2^ after 10 min.

#### 2.4.2. Electrothermal CCGA Prepared with Different Suspension Concentrations

In order to clearly observe the distinction among the CCGA prepared with different suspension concentrations, a higher power density of 2000 W m^−^^2^ was applied in this section. Electrification was running for 20 min then the power was turned off and cooled for 20 min, in which their surface temperature was recorded. And the voltage and the electric current was also recorded to calculate the resistance between two electrodes during the electrification, to evaluate the electric heating stability. As well as that the temperature distribution on the surface was analyzed by using the thermal infrared imager.

#### 2.4.3. Electrothermal CCGA Prepared with Various NFC Amounts

Electrification on CCGA 40, CCGA 60, and CCGA 80 was carried out under the power density from 500 to 3000 W m^−2^ at intervals of 500 W m^−2^ for 20 min follow cooling for 25 min, the temperature and the resistance between two electrodes was also recorded. The resistance drop rate at the moment of power off was used to evaluate the electric heating stability.

## 3. Results and Discussion

### 3.1. Effect of Ultrasonic Power and Time on the Performance of CCGA

**Figure 3:**
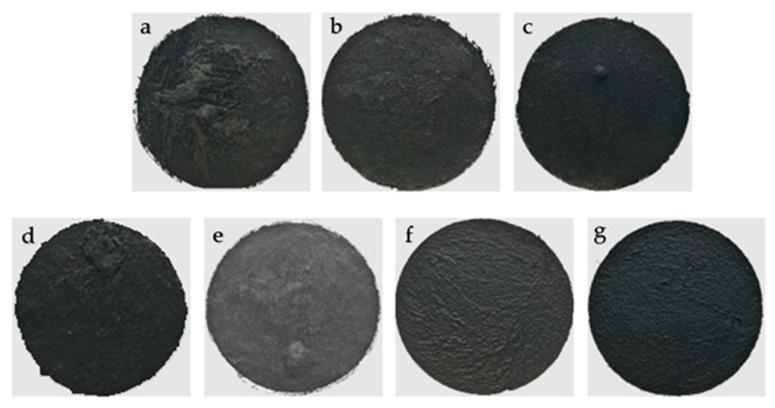
Photographs of CCGA prepared via various ultrasonic conditions of (**a**) 600 W, 40 min; (**b**) 800 W, 40 min; (**c**) 1000 W, 40 min; (**d**) 1000 W, 10 min; (**e**) 1000 W, 30 min; (**f**) 1000 W, 50 min; (**g**) 1000 W, 70 min.

**Figure 4:**
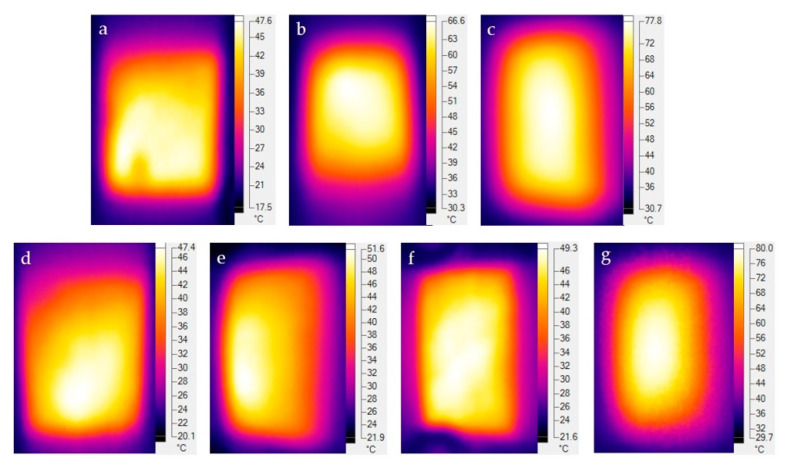
Infrared thermogram of CCGA prepared via various ultrasonic conditions of (**a**) 600 W, 40 min; (**b**) 800 W, 40 min; (**c**) 1000 W, 40 min; (**d**) 1000 W, 10 min; (**e**) 1000 W, 30 min; (**f**) 1000 W, 50 min; (**g**) 1000 W, 70 min.

Ultrasonic dispersion can promote the uniformity of the dispersibility of NFC and nano-carbon materials in aqueous suspension [41], and thus can improve the electric heating performance of electrothermal CCGA. After the comparison in Figure 3a–c, the CCGA prepared at 1000 W (Figure 3c) had better surface smoothness and flatness, which exhibited a more uniform electric heating temperature (Figure 4) under the power density of 1000 W m^−2^ after electrified for 10 min. Under the fixed ultrasonic power of 1000 W, surface smoothness and regularity of the CCGA became better with the extension of ultrasound time from 10 min to 70 min, as shown in Figure 3d–g. Obviously, the surface wrinkle area gradually became smaller. Combined with the analysis of the infrared thermogram in Figure 4d–g, results indicated that the extension of the ultrasonic time can increase the temperature uniformity on the electrothermal CCGA. And the maximum surface temperature also increased significantly. In which, the preparation process with 1000 W and 50 min was more reasonable and was employed in the subsequent experiment.

### 3.2. SEM Morphology Analysis

**Figure 5:**
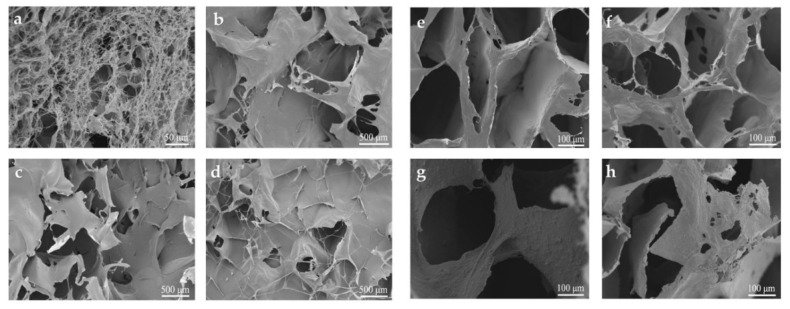
SEM image on upper surface of the aerogel (**a**) pure NFC, (**b**) CCGA 40, (**c**) CCGA 60, (**d**) CCGA 80 and SEM analysis at cross-section of the aerogel (**e**) pure NFC, (**f**) CCGA 40, (**g**) CCGA 60, (**h**) CCGA 80.

Aa shown from SEM analysis in Figure 5a–d, pure NFC aerogel (Figure 5a) shows a porous structure consisting of microfibrillar networks, and the fibrils coagulated to form a small number of nanosheets [44,45], while the surface pores of CCGA decreased after added with GP. During the freeze-drying process, MWCNTs and GP easily entangle with NFC, which would affect the nucleation and growth of ice crystals when forming aerogel [46]. SEM results on the cross-section of aerogel shown in Figure 5e–h indicate a clear and typical three-dimensional porous network structure. The interconnected pores formed in both pure NFC aerogel (Figure 5e) and the CCGA (Figure 5f–h). The network structure also formed many tiny pores on the pore wall. What’s more, NFC and GP can synergistically improve the dispersibility of MWCNTs in the suspension [47]. The dispersion is due to interfacial interactions among the oxygen-containing groups on the nano-carbon materials and cellulose material [48]. Therefore, the highly interconnected three-dimensional cellular networks was obtained, which may be responsible for the outstanding mechanical properties of the aerogel [46]. It is worth noting that the abundant porous structure is conducive to the heat exchange with the environment in the electric heating process [21,22,23].

### 3.3. XRD Analysis

**Figure 6:**
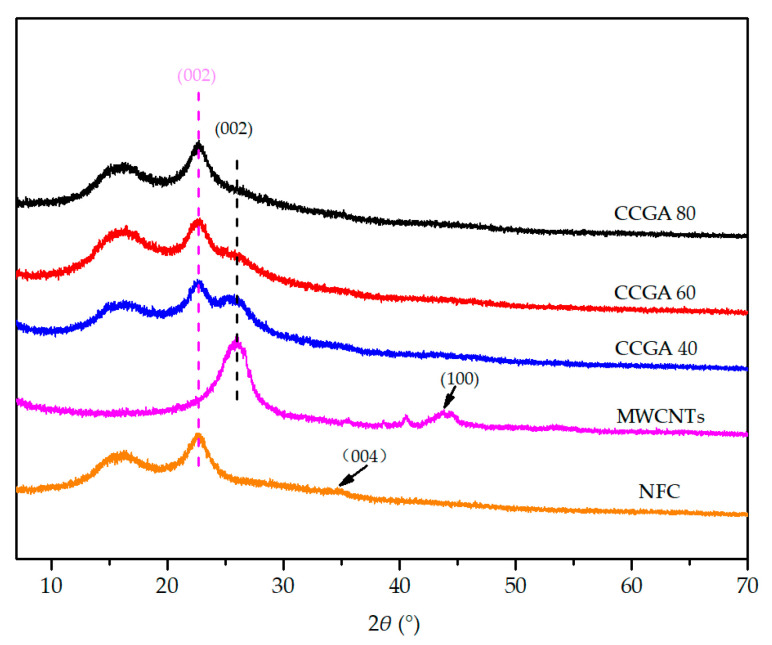
XRD patterns of the different aerogels.

XRD diffraction patterns of different aerogels are shown in Figure 6. Pure NFC aerogel has sharp and narrow diffraction peaks at 2*θ* = 22.7°, corresponding to the (002) crystal plane of cellulose I [49]. There are two overlapping and weaker peaks at approximately 14.8° and 16.9°, corresponding to the structure of cellulose I [50,51], and there is an indistinct (004) crystal plane diffraction peak located near 34.8° [50,51], indicating that it contains a semi-crystalline structure. In addition, a sharp and wide peak at 25.9° can be seen in the XRD diffraction pattern of MWCNTs aerogel, which corresponds to the diffraction peak of (002) lattice plane [52], and a weaker (100) plane diffraction peak appears at 43.8° [53]. The characteristic peak located at 14.8°, 16.9° and 22.7° became gradually stronger as increasing the NFC content in CCGA. In the meantime, the intensity of the typical (002) lattice plane diffraction peak located near 25.9° becomes weak, which cannot be found distinctly as NFC content exceeding 80 wt.%. These results indicated the addition of NFC exceeding 80 wt.% can effectively avoid agglomeration of nano-carbon materials, which is similar to early reports [54]; both nano-carbon materials and NFC was homogeneously dispersed to form a three-dimensional porous structure.

### 3.4. FT-IR, pH, and Electrical Conductivity Analysis

**Figure 7:**
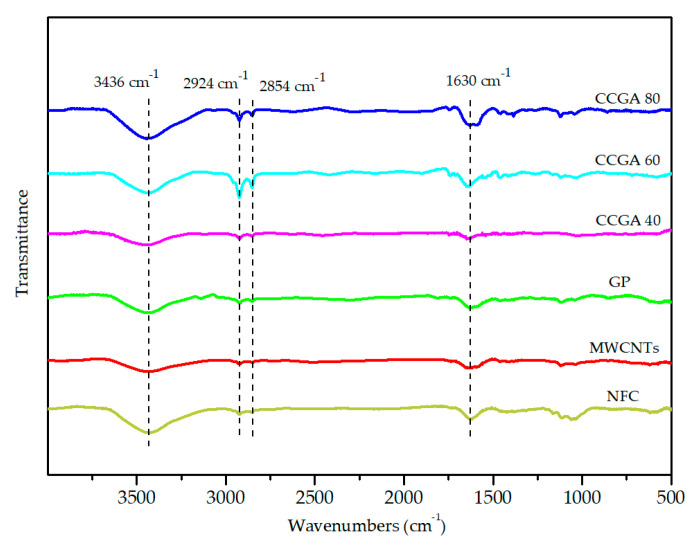
FT-IR spectra of the different aerogels.

The FT-IR spectra of MWCNTs and GP in Figure 7 shows two weak peaks at 2924 cm^−1^ and 2854 cm^−1^, which is caused by the methylene group (–CH_2_–) in the crystal lattice, and there is a broad peak near 3432 cm^−1^, linking to the –OH group on the edge of the nano-carbon materials [28,55]. The peak around 1630 cm^−1^ is the C = C stretching vibration absorption peak [56,57]. Pure NFC aerogel has a significant –OH stretching vibration peak around 3436 cm^−1^, while the characteristic peak at 2800–3000 cm^−1^ is attributed to –CH_2_–. The characteristic peak at 1650–1630 cm^−1^ corresponds to the water absorbed by the NFC [58]. The peak at 1170–1082 cm^−1^ has a relation with the C–O–C binding group [59]. With the increase of NFC content, –OH stretching vibration peak near 3436 cm^−1^ became stronger, and the characteristic peak of –CH_2_– and C = C groups in CCGA 80 was still distinctly. Obviously, the surface of nano-carbon materials and NFC also contains a certain of functional groups (such as –OH and other oxygen-containing functional groups) shown in Figure 7. The electrostatic repulsive forces induced from the oxygen-containing groups and the hydrophobic interaction between NFC and MWCNTs facilitate the dispersion of MWCNTs in NFC suspension [33], which is consistent with the result of SEM analysis. MWCNTs with oxygen-containing groups can be also seen as the functionalized CNTs, which can favor the dispersion, assembly and reinforcement in the substrate [60].

Furthermore, as seen in Table 1, measurement results on the pH of the suspension with various NFC contents demonstrate that all the suspension were alkalescent even changing the content of NFC or the nano-carbon materials. So, the NFC can remain negative potential on its surface in the suspension, and form electrostatic repulsive forces to disperse the nano-carbon materials [41,42,61]. As well as electrical conductivity (EC) of the suspension shown in Table 2, gradually increasing when adding the content of NFC, further indicate the negatively charged groups on the NFC surface [62]. Therefore, the NFC/MWCNTs/GP suspension and the CCGA prepared with a moderate amount of NFC could gain good uniformity of structure.

### 3.5. Thermal Properties of the Aerogels

**Figure 8:**
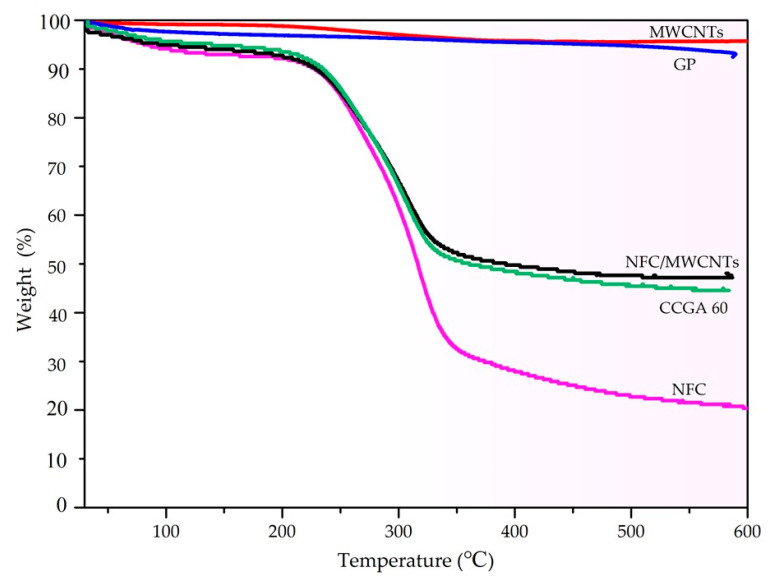
Thermogravimetric (TG) cures of the aerogels.

The thermal stability (Figure 8) of the aerogel can be introduced to appraise the electric heating stability from another perspective. The mass loss of NFC in the range of 30–125 °C mainly results from the remove of the water absorbed by the NFC [63], and then the second major mass loss of NFC in the temperature range of 225–350 °C is mainly due to the breakage of the backbone of NFC to form low molecular weight volatile chemicals. The thermal stability of CCGA 60 (NFC content was 60 wt.%) is significantly better than that of pure NFC aerogel, shown from the second mass loss process of the CCGA, which was mainly attributed to the decomposition of NFC [52]. The mass loss at 580 °C of pure GP and MWCNTs is only about 7% and 5%, respectively, indicating that the thermal stability of the used MWCNTs is slightly better than the used GP. Therefore, it can be seen that the thermal stability of NFC/MWCNTs aerogel (NFC content was 60 wt.%) is better than that of the CCGA, but there is only a small difference. Results show that the CCGA with a three-dimensional porous structure has good thermal stability. Under the synergy effect of MWCNTs and GP, the CCGA has almost no quality loss except for the loss of the absorbed moisture below 150 °C. Consequently, the designed electrothermal CCGA can keep its working stability below 150 °C. 

### 3.6. Electric Heating Performance of CCGA

#### 3.6.1. Electric Heating Performance of CCGA Prepared with Different Suspension Concentrations

**Figure 9:**
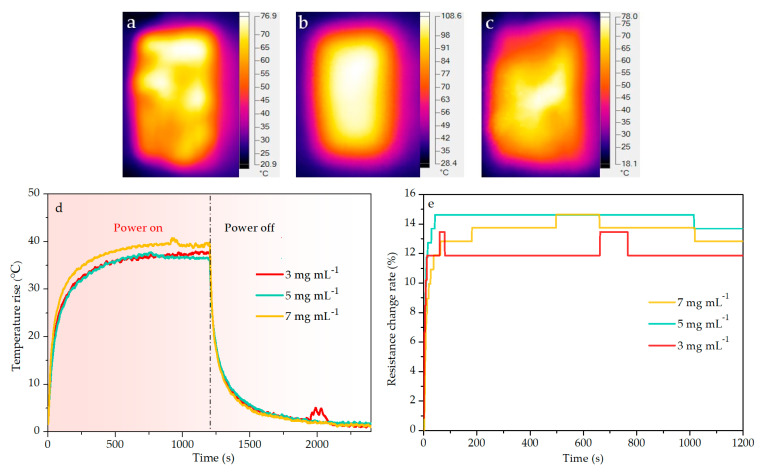
Infrared thermogram of the electrothermal CCGA: (**a**) 3–CCGA, (**b**) 5–CCGA, (**c**) 7–CCGA; (**d**) time–temperature rise curve of different electrothermal CCGA; (**e**) resistance change rate and time relationship during the electrification for 20 min under the power density of 2000 W m^−2^.

Nano-carbon materials have excellent electrical conductivity and thermal conductivity [5,6,7,8], as well as lightweight and good chemical stability, which could endow the CCGA with certain advantages in the application of electrothermal materials. The infrared thermogram in Figure 9 shows similar surface temperature distribution on the all developed electrothermal CCGA with various concentrations during the electrification process. It can be found from Figure 9d that the electrothermal CCGA showed similar temperature rise law to the previously reported GP or CNTs-based electrothermal materials [42,43,64]. Under the applied power density of 2000 W m^−2^, all the electrothermal CCGA had a rapid heating rate and reached the maximum steady-state temperature rise in about 500 s. Even the concentration was as low as 3 mg mL^−1^, the CCGA had the similar temperature rise and heating rate, signifying that the density of CCGA can be reduced to elevate its porosity. The similar stabilized temperature rise existed in all electrothermal CCGA.

It can be found in Figure 9e that there was a slight distinction among the resistance change rate of the electrothermal CCGA. Little higher resistance change rate occurred in the electrothermal CCGA prepared at higher concentration. When the input voltage was fixed, the lower the resistance was, the higher power was [65]. It can be also concluded from the resistance change rate that the real power at the running state was higher than the set power. As seen from Figure 9d, the temperature rise was about 40 °C at the 7 mg mL^−1^, which was lower than the NFC–GP membrane under the same power density of 2000 W m^−2^ [43]. However, the resistance change rate of the electrothermal CCGA (nearly 15% at the 7 mg mL^−1^) was much less than that of the NFC-GP membrane (about 37% while the addition of GP was 50 wt.%) under the same power density of 2000 W m^−2^ [43]. Moreover, the resistance change rate increased rapidly in the initial with the increase of the temperature, while it stabilized in the following process. The results illuminate that the electrothermal CCGA have relatively stable electric heating performance.

#### 3.6.2. Electric Heating Performance of CCGA Prepared with Different NFC Contents

**Figure 10:**
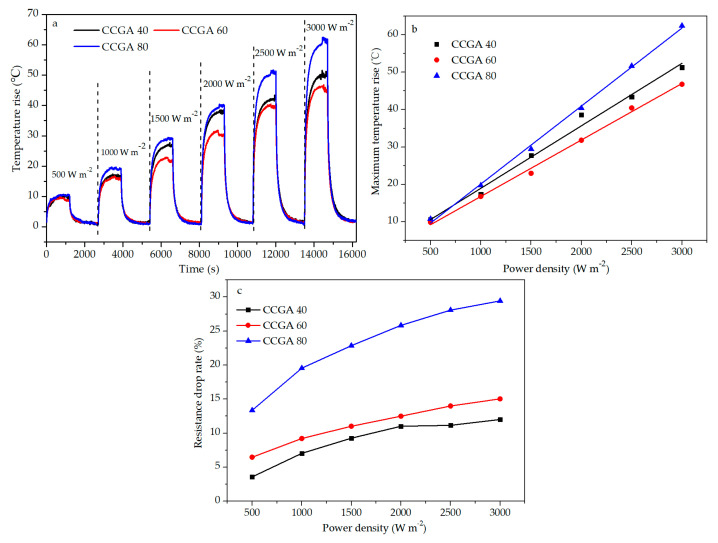
(**a**) Time-temperature rise curve of electrothermal CCGA with different NFC contents, (**b**) fitting curve between maximum temperature rise and power density of electrothermal CCGA with different NFC contents, (**c**) relationship between resistance drop rate-power density of electrothermal CCGA with different NFC contents.

Figure 10a shows the time-temperature rise relationship in the operation of the on–off power cycle under continuous power density (500–3000 W m^−2^). It needed more time to reach a steady state when applied with higher power density. There is a linear correlation between power density and temperature rise shown in Figure 10b even incorporated with the different NFC amounts. Compared with the previous report on the electric heating membrane [43], the temperature rise of the electrothermal CCGA with 80 wt.% NFC was as low as 62.0 °C under the high power density of 3000 W m^−2^.

As seen in Table 3, the greater the power density was applied, the higher the resistance drop rate of the electrothermal CCGA was, indicating the positive relationship between them, as well as the drop rate and the temperature rise. The resistance change trend is similar to previous reports [43]. Figure 10c shows the resistance drop rate will increase with the addition of NFC content. It could be attributed to the low stability of the NFC, which has been illuminated in the above thermal analysis.

When the set power was 1.8 W (3000 W m^−2^), the maximum equilibrium temperature of electrothermal CCGA 80 was about 85.5 °C (room temperature 23.5 °C) shown in Figure 10a. The electric heating performance of this electrothermal CCGA (the set power of 0.6 W, maximum steady-state temperature of 43.2 °C) under 1000 W m^−2^ was close to that of the GP foam coated with Ag (0.36 W, 38.0 °C) [66]. Consequently, this electrothermal CCGA exhibits a relatively steady and efficient electric heating performance. Importantly, its porous structure is conducive to enhancing its heating surface, which endows itself with high heat exchange efficiency [23].

## 4. Conclusions

In this work, a porous electrothermal CCGA was presented. NFC as dispersant and binder assembles the MWCNTs and GP to manufacture highly porous CCGA via freeze-drying. The homogeneous surface of CCGA can be obtained by controlling ultrasonic conditions (time and power). Similar uniform temperature distribution existed on the surface of electrothermal CCGA with various sonication conditions. The concentration of the NFC/MWCNTs/GP suspension determines the density and porosity of the CCGA and slightly affects its electric heating performance. As well as adding the amount of NFC, the resistance drop rate will increase, which results in the difference of temperature rise of the electrothermal CCGA with different NFC amounts, especially under the high power density. The results demonstrate that CCGA 40 has higher electric heating stability in this study. The porous structure would be conducive to the heat exchange of electrothermal CCGA. Therefore, the prepared porous electrothermal CCGA based on NFC can be used as a potential lightweight substrate for efficient electrothermal devices.

## Figures and Tables

**Table 1:** pH of the suspension with different NFC contents.

Content of NFC	pH of Group 1	pH of Group 2	Average pH
**40 wt.%**	8.29	8.43	8.36
**60 wt.%**	8.16	8.28	8.22
**80 wt.%**	8.23	8.26	8.25
**100 wt.%**	8.24	8.24	8.24

**Table 2:** Electrical conductivity (EC) of the suspension with different NFC contents.

Content of NFC	EC of Group 1 (μs cm^−1^)	EC of Group 2 (μs cm^−1^)	Mean EC (μs cm^−1^)
**40 wt.%**	211	188	199.5
**60 wt.%**	367	388	377.5
**80 wt.%**	496	602	549.0
**100 wt.%**	552	538	545.0

**Table 3:** Maximum temperature rise and resistance drop rate of electrothermal CCGA with different NFC contents under different power density.

Index	Content of NFC	Power Density (W m^−2^)					
		500	1000	1500	2000	2500	3000
**Maximum temperature rise** **(°C)**	40 wt.%	10.55	17.35	27.75	38.60	43.40	51.60
	60 wt.%	9.85	16.75	22.95	31.80	40.45	46.80
	80 wt.%	10.75	19.70	29.45	40.4	51.7	62.00
**Resistance drop rate (%)**	40 wt.%	3.58	7.04	9.26	11.02	11.15	12.01
	60 wt.%	6.47	9.22	11.02	12.49	13.99	15.04
	80 wt.%	13.36	19.55	22.85	25.84	28.07	29.43
